# Change in β_2_-agonist use after severe life events in adults with asthma: A population-based cohort study: Life events and bronchodilator usage among adults with asthma

**DOI:** 10.1016/j.jpsychores.2017.07.003

**Published:** 2017-09

**Authors:** Raija Lietzén, Pekka Virtanen, Mika Kivimäki, Jyrki Korkeila, Sakari Suominen, Lauri Sillanmäki, Markku Koskenvuo, Jussi Vahtera

**Affiliations:** a Department of Public Health, University of Turku, Turku, Finland; b School of Health Sciences, University of Tampere, Tampere, Finland; c Department of Public Health, Clinicum, Faculty of Medicine, University of Helsinki, Helsinki, Finland; d Department of Epidemiology and Public Health, University College London Medical School, London, United Kingdom; e Department of Psychiatry, University of Turku and Harjavalta Hospital, Satakunta Hospital District, Harjavalta, Finland; f Department of Public Health, University of Skövde, Skövde, Sweden; g Department of Public Health, University of Turku and Turku University Hospital, Turku, Finland

**Keywords:** Asthma, Cohort study, Generalized estimating equation method, Life events, Short-acting β_2_-agonists, Stressful life events

## Abstract

**Objective:**

This prospective, population-based cohort study of 1102 Finnish adults with asthma, examined whether exposure to stressful life events is associated with the intensity of usage of inhaled short-acting β_2_-agonists.

**Methods:**

Survey data was collected by two postal questionnaires. Baseline characteristics were obtained in 1998 and data on 19 specific stressful events (e.g. death of a child or spouse or divorce) within the six preceding months in 2003. Exposure to life events was indicated by a sum score weighted by mean severity of the events. Participants were linked to records of filled prescriptions for inhaled short-acting β_2_-agonists from national registers from 2000 through 2006. The rates of purchases of short-acting β_2_-agonists before (2000 − 2001), during (2002 − 2003) and after (2004–2006) the event exposure were estimated using repeated-measures Poisson regression analyses with the generalized estimating equation.

**Results:**

Of the 1102 participants, 162 (15%) were exposed to highly stressful events, 205 (19%) to less stressful events. During the 7-year observation period, 5955 purchases of filled prescription for inhaled short-acting β_2_-agonists were recorded. After exposure to highly stressful events, the rate of purchases of β_2_-agonists was 1.50 times higher (95% confidence interval (CI): 1.05, 2.13) than before the stressful event occurred. Among those with low or no exposure to life events, the corresponding rate ratios were not elevated (rate ratio 0.81, 95% CI: 0.66, 0.99 and 0.95, 95% CI: 0.83, 1.09 respectively).

**Conclusion:**

An increase in β_2_-agonist usage after severe life events suggests that stressful experiences may worsen asthma symptoms.

## Highlights

• Bronchodilator purchases increased in adults with asthma following stressful events.
• Stressful life events may worsen asthma symptoms.
• Results highlight the importance of psychosocial stress in management of asthma.

## 1 Introduction

Asthma is a chronic intermittent inflammation of the large airways [1], [2] with the reported population prevalence varying from 2% in Estonia to 21% in Australia [3]. Its prevalence is increasing in many countries [4].A large number of studies on the biological risk factors for asthma morbidity have found evidence of the etiological importance of respiratory infections, allergens, air pollutants, and tobacco smoke [5], [6]. Recently, the role of psychosocial stress as a contributor of asthma morbidity has gained increased attention [7], [8]. Stress is considered to affect the exacerbation of asthma through multiple immune, endocrine, neural, and behavioural processes [9], [10]. Stress also accentuates the individual's immune response and induces changes in inflammatory processes in the airways [11].

Some longitudinal cohort studies suggest an association between negative stressful life events in the family and elsewhere and asthma onset [12], [13], [14], while others have not found any association [15]. The most recent study suggests that both work stress and family related life events are positively associated with asthma in women [16]. In addition, life events may lead to a worsening of asthma among asthmatic adults [17], [18], [19]. Life events were risk factors for hospital admissions due to asthma in a population based study [18] and hospital admission for acute severe asthma in a case-control study [19]. Exposure to community violence was associated with both hospital admission and emergency department visits due to asthma in a study among adults [17] while long-lasting stress, at least at work, was not associated with severe asthma exacerbations leading to hospitalization or death [20]. The previous studies scope on hospitalizations, which represent serious exacerbations of asthma that occur at times in most patients and have decreased during the last years [21].

Prescriptions of asthma medication may provide a way of assessing day-to-day variation in everyday asthma symptoms in relation to increase in life stress. To date, however, the association between recent stressful life events and usage of inhaled bronchodilators has not been studied in asthmatic adults in spite of the fact that a cornerstone of successful treatment for asthma is self-management. It includes the perception of asthma symptoms and the use of prescribed medication. The aim of medication is to control the disease and to prevent its exacerbation. Inhaled corticosteroids, in combination with or separate from long-acting β_2_-agonists as anti-inflammatory drugs, play a major role in this treatment. In addition, inhaled short-acting β_2_-agonists are used for bronchodilation and protection against bronchoconstriction and as quick-relief drugs for asthma symptoms. [22], [23]

In this prospective study, we hypothesized that people with asthma inhale short-acting β_2_-agonists when they are symptomatic and that high exposure to recent stressful life events would be associated with worsening symptoms, resulting in increased purchase of inhaled short-acting β_2 −_-agonist medication. To examine this, we used data of filled prescriptions for asthmatic persons before, during, and after exposure to recent stressful life events over a seven-year observation period. To the best of our knowledge, no large-scale, general population studies have examined changes in purchases of short-acting β_2 −_-agonists following recent stressful life events among adults with diagnosed asthma.

## 2 Method

### 2.1 Study design and participants

**Fig. 1:**
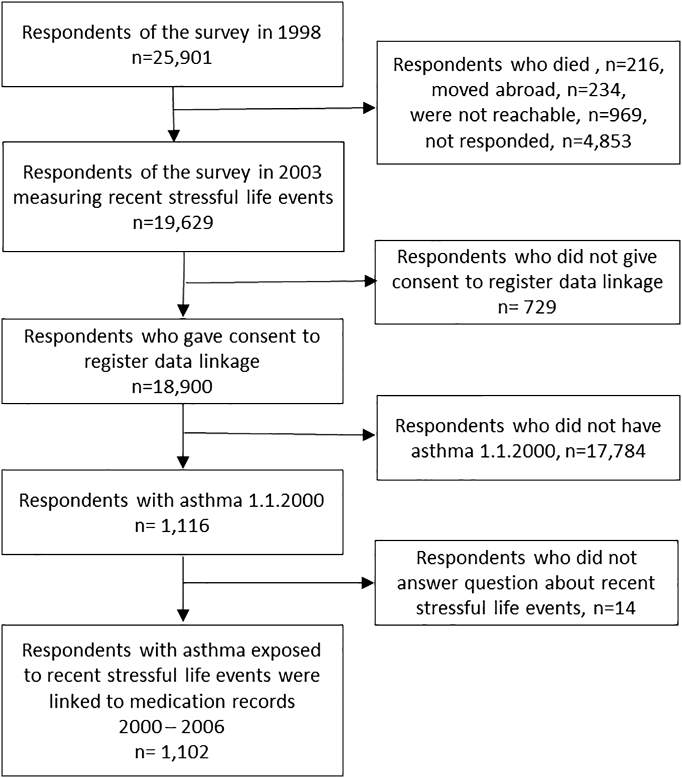
Sample selection of subjects with asthma exposed to recent stressful life events and linked to medication records 2000–2006, the Health and Social Support Study in Finland, 1998–2006. The identification of a respondents having asthma was based on national registers.

The Health and Social Support Study, a longitudinal cohort study, is based on a representative sample of the Finnish population in the age groups: 20–24, 30–34, 40–44, and 50–54 years at baseline [24]. The baseline postal survey was conducted in 1998, and a total of 25,901 respondents returned the questionnaire. Of them, 19,629 respondents (80% of those eligible) participated in a follow-up survey 5 years later in 2003 and 18,900 (96%) consented to the use of their recorded health information from the Finnish national registers. For this study, we selected all participants of the follow-up survey with asthma (*n* = 1102, 73% women) at the beginning of the 7-year observation window from January 1, 2000 onward who had provided information on the occurrence of new life events within the preceding six months in the follow-up survey. (Fig. 1) The study was approved by the Turku University Hospital Ethics Committee. All participants signed an informed consent form.

### 2.2 Participants with asthma

We used the unified personal identification code system, covering all Finnish citizens, to link and obtain records from three administrative and comprehensive Finnish national health registers to identify individuals with asthma and their purchases of prescribed asthma medications.

The identification of a participant having asthma at the beginning of the follow-up was based on the clinical diagnosis of the treating physician in the records of the Drug Reimbursement Register of the Social Insurance Institution (SII) of Finland [25] and/or the Hospital Discharge Register of the National Institute for Health and Welfare. We used the Drug Reimbursement Register of the SII of Finland containing information on persons entitled to special reimbursement for certain chronic diseases, such as asthma. Patients who apply for special reimbursement must attach a detailed medical certificate prepared by the treating physician, who also provides data to confirm the diagnosis. The application is then reviewed by a physician in the SII to determine whether the uniformly defined requirements for the disease are met. From this register, participants were defined as asthma cases if they were for the first time recorded in the Central Drug Register as eligible for asthma treatment before the start of the follow-up in January 1, 2000. Moreover, we used prescription data to assess the beginnings of medical treatment for asthma. In Finland, the National Social Insurance Scheme at the SII provides basic reimbursement for all filled outpatient prescriptions that are recorded in the Drug Prescription Register according to the World Health Organization's Anatomical Therapeutic Chemical (ATC) Classification. The date of purchase is also recorded. We identified all participants with two or more prescriptions for drugs for obstructive airway diseases (ATC code R03) in 1998 and 1999 (the two years preceding the beginning of the follow-up) by using the day of the first purchase as an indicator of prevalent asthma. Finally, we obtained data from the Hospital Discharge Register of the National Institute for Health and Welfare, which includes records on all inpatient hospital admissions [26]. This register is comprised of countrywide information on virtually all hospitalizations. All participants discharged from hospitals with the main diagnosis ICD-10 J45 (asthma) within the two years preceding the beginning of the follow-up were also defined as asthma cases.

### 2.3 Assessment of filled prescription for asthma medication during the 7-year follow-up

In Finland, inhaled asthma medications are only available by prescription. The National Health Insurance Scheme, run by the SII of Finland, provides prescription drug coverage for all (~ 5.5 million) community-dwelling residents of Finland. All reimbursed prescriptions are registered in the Drug Prescription Register managed by the SII [25]. For each drug, the dispensing date and the World Health Organization Anatomical Therapeutic Chemical (ATC) code are recorded. We derived the date and the ATC classification code of purchases of inhaled β_2_-agonists and corticosteroids during a seven-year observation period covering the years 2000 to 2006 from the Drug Prescription Register. For observation, we determined the number of purchases for inhaled short-acting β_2_- agonists (ATC R03AC02, R03AC03 R03AC04) and combinations of short-acting β_2_-agonists with anticholinergics (ATC R03AK03, R03AK04).

Because the need for short-acting β_2_-agonists may depend on the simultaneous usage of inhaled anti-inflammatory medication, we determined the number of purchases for inhaled corticosteroids (ATC R03BA01, R03BA02, R03BA05) and long-acting β_2_-agonists (ATC R03AC12, R03AC13) in non-combination inhalers and fixed dose combination inhalers of inhaled corticosteroids and long-acting β_2_-agonists (ATC R03AK06, R03AK07). The number of purchases in every year of observation was handled as a categorical factor in the model.

### 2.4 Recent stressful life events

We measured the occurrence of recent stressful life events in the follow-up survey conducted in 2003 by using 19 life events from a list of 21 events [27], [28]. The excluded events (i.e., “illness causing work disability of over 21 days” and “disability retirement”) might have been a consequence of asthma exacerbation (see Appendix 1). For the timing of each event, the questionnaire included four response alternatives (never, within the previous 6 months, within the previous 5 yrs. and > 5 yrs. ago), and the respondents were instructed to select only one of them. The focus of this study is in recent events, those that had occurred during the previous six months. We assessed the level of exposure to recent life events for each individual by calculating a cumulative mean sum score weighted by the average severity of the event (for weights, see Vahtera et al. [28]). The cumulative severity ratings ranged from 2.74 to 24.64. Those respondents with a 0 (zero) score were defined as having no exposure. Those who had been exposed were divided into two groups using the median of the cumulative severity score as the cut-off point (low exposure < 5, high exposure ≥ 5).

As shown in Appendix 1, only a small proportion of participants had reported the corresponding event in the 1998 survey (e.g. 14% of those who were victims of violence in 2003 reported to have been victims in 1998 also). Events with highest recurrence - breakup of long-term friendship (43%), severe financial difficulties (40%) and death of a close relative (35%) - were rated much less severe and, thus, had a lower weight in the cumulative mean sum score.

### 2.5 Baseline characteristics

Baseline characteristics, measured in the baseline survey in 1998 before exposure to the life events included socio-demographic variables – sex, age group, marital status, and level of education – behaviour-related health risks, sensitivity to stress and depression. The behaviour-related health risks were smoking (never/ex/current), high alcohol intake (≥ 175 g of alcohol for women and ≥ 263 g of alcohol for men per week) [29], obesity (Body Mass Index (BMI) ≥ 30 kg/m^2^) and physical inactivity (the Metabolic Equivalent Task index < 2 MET-hours/day) [30]. Individual differences in sensitivity to stress were measured by general feelings of stressfulness in daily life [28], [31]. The mean scores of the scale were divided with tertiles, with the highest third used as an indicator of sensitivity to stress. Depression, a potential mediator between a stressful life event and asthma, was assessed using the Beck Depression Inventory (sum score > 18) [32] and the Drug Prescription Register [≥ 1 antidepressant (ATC-code N06A) purchases in 1998]. Participants showing depression in any of these measurements were classified as cases of pre-existing depression.

### 2.6 Statistical analysis

The association between background variables (demographics, health-related factors, and psychological factors) (measured in 1998) and recent stressful life-event exposure (measured in 2003) categories were studied using the Pearson's chi-squared tests.

For the analyses, we divided the seven-year follow-up time into three periods in relation to the timing of the life-event exposure: ‘before’ (i.e., year − 3 and − 2, 2000–2001), ‘during’ (i.e. year − 1 and 0, 2002–2003) and ‘after’ (i.e. year + 1 to + 3, 2004–2006). We applied a repeated-measures Poisson regression analysis with the generalized estimating equation (GEE) method and autoregressive correlation structure [33]. The GEE takes into account the correlation of annual medication purchases within persons, and is not very sensitive to missing cases at repeated measurements. We first examined the purchases of inhaled short-acting bronchodilators by baseline characteristic by calculating the mean rates of purchases over the seven-year observation period for each characteristic.

We used contrasts to estimate the rate ratios and their 95% confidence intervals (95% CI) during and after the exposure compared with the period before the exposure - within each exposure group using models including the interaction term “stressful life-event exposure*period”. The rate ratios for purchases of inhaled short-acting β_2_-agonists were calculated in the three time periods around the life-event exposure. The analyses were adjusted for all baseline characteristics. An additional adjustment was made for inhaled anti-inflammatory medication. In order to examine whether changes in purchases of inhaled short-acting β_2_-agonists following exposure to life events varied between the subgroups (e.g. by sex, age group, education, behaviour-related health risks, depression or sensitivity to stress) we calculated the rate ratios for each subgroup by using the same model. All tests were 2-tailed.

All analyses were performed using the SAS Enterprise Guide 6.100 (6.100.0.2870) statistical software (SAS Institute Inc., Cary, NC, USA, 2013).

## 3 Results

### 3.1 Characteristics of the study population

The sample included 296 (27%) men and 806 (73%) women with prevalent asthma at baseline. Of these individuals, 367 (33%) reported an occurrence of new recent stressful life events in the follow-up survey. The events reported most often were: ‘major increase in marital problems’ (*n* = 83), ‘severe financial difficulties’ (*n* = 77) or/and ‘death of another close relative’ (*n* = 60). (Appendix 1). Table 1 shows the associations between the severity of life events exposed (no/low/high) and the characteristics of participants; a young age, current or ex smoking, and physical activity were associated with high life-event exposure.

**Table 1:** Characteristics of the participant at baseline in 1998 by recent stressful life-event exposure levels in 2003 and annual mean rates for purchases of inhaled short-acting β_2_-agonists during 2000–2006, the health and social support study in Finland, 1998–2006.

	Level of life-event exposure										
	All participants		No		Low		High			Purchases 2000–2006	
	No.	%	No.	%	No.	%	No.	%	*P* Value^a^	Mean rate^b^	*P* Value^c^
Total	1102	100	73	66	205	19	162	15			
Sex									0.54		0.96
Men	296	27	200	27	58	28	38	23		77.6	
Women	806	73	535	73	147	72	124	77		77.1	
Age group									< 0.001		0.20
20–24	242	22	135	18	57	28	50	31		64.1	
30–34	239	22	145	20	53	26	41	25		79.2	
40–44	252	23	171	23	48	23	33	20		71.5	
50–54	369	33	284	39	47	23	38	24		88.5	
Occupational education									0.070		0.035
University	177	16	129	18	29	14	19	12		59.7	
College	335	31	211	29	74	37	50	31		69.4	
Vocational school	237	22	158	22	34	17	45	28		75.0	
Basic	336	31	225	31	64	32	47	29		94.5	
Marital status									0.015		0.24
Single/divorced/widowed	326	30	199	27	65	32	62	38		85.4	
Married/cohabiting	775	70	535	73	140	68	62	62		73.5	
Smoking									0.003		< 0.001
Never-smoker	449	45	328	49	72	39	49	32		60.4	
Ex-smoker	296	29	186	28	56	30	54	36		66.1	
Current smoker	266	26	161	24	57	31	48	32		124.8	
Physical inactivity									0.008		0.41
No	826	75	532	73	171	83	123	76		84.2	
Yes	270	25	198	27	34	17	38	24		74.2	
Obesity (BMI ≥ 30)^d^									0.53		0.007
No	921	84	609	83	173	84	139	87		68.8	
Yes	175	16	122	17	32	16	21	13		124.0	
High alcohol intake^e^									0.43		0.72
No	1046	95	701	96	193	94	152	94		77.0	
Yes	53	5	31	4	12	6	10	6		84.1	
Depression									0.36		0.036
No	981	89	659	90	183	89	139	86		73.5	
Yes	121	11	76	10	22	11	23	14		107.4	
Sensitivity to stress									0.066		0.22
No	720	66	496	68	127	62	97	60		72.4	
Yes	374	34	232	32	77	38	65	40		86.3	

Abbreviations: BMI, body mass index.

a *P* value for difference between the exposure groups (Pearson's chi-squared test).

b Annual average purchases of inhaled short-acting β_2_-agonists per 100 person years derived from Poisson regression generalized estimating equation (GEE) analysis for covariate.

c *P* value from Poisson regression GEE analysis for mean rates.

d BMI was calculated as weight (kg)/height (m^2^).

e High alcohol intake refers to consumption of > 175 g/week for women and > 263 g/week for men.

### 3.2 Purchases of inhaled short-acting β_2_-agonists

During the seven-year observation period, 5955 purchases of inhaled short-acting β_2_-agonists were recorded for the participants. A high annual purchase rate was observed among smokers, as well as those with depression, obesity and those with a basic level of education (Table 1).

### 3.3 Inhaled short-acting β_2_-agonists and recent stressful life events

Table 2 shows the rate ratios for purchases of inhaled short-acting β_2_-agonists in the time periods during (year − 1 and 0) and after (year + 1 to + 3), compared with the period before (year − 3 and − 2), exposure to stressful life events adjusted for the baseline characteristics (demographics, health risk behaviours, depression and sensitivity to stress) and, additionally, for inhaled anti-inflammatory medication. Among those with no exposure to recent stressful life events, the rate of purchases did not significantly vary between the time periods. Among participants who had encountered highly stressful recent life events, the purchases of inhaled short-acting β_2_-agonists increased by 46% during the life event exposure and 52% after the exposure compared with the pre-exposure levels. This rate ratio remained unchanged after taking into account adjustments for all baseline characteristics. Additionally, adjustment for inhaled anti-inflammatory medication only slightly attenuated the association (*p* < 0.06). Among participants who had low exposure to recent stressful events, no increase between periods existed.

**Table 2:** Rate Ratios for Purchases of Inhaled Short-Acting β_2_-Agonists Comparing Different Time Periods According to Life-Event exposure, The Health and Social Support Study in Finland, 1998–2006.

Adjustment level of stressful life-event exposure	Time in relation to life-event exposure			
	During^a^ vs before^c^		After^b^ vs before^c^	
	RR^d^	95% CI	RR^d^	95% CI
Unadjusted				
No exposure	0.98	0.88, 1.09	0.94	0.82, 1.07
Low exposure	0.84	0.75, 0.95	0.89	0.72, 1.10
High exposure	1.46	1.12, 1.90	1.52	1.06, 2.16
Baseline adjusted^e^				
No exposure	0.99	0.88, 1.11	0.95	0.83, 1.09
Low exposure	0.84	0.74, 0.96	0.81	0.66, 0.99
High exposure	1.47	1.12, 1.92	1.50	1.05, 2.13
Additionally adjusted^f^				
No exposure	0.97	0.85, 1.10	0.92	0.79, 1.07
Low exposure	0.79	0.69, 0.91	0.74	0.62, 0.88
High exposure	1.43	1.09, 1.87	1.43	0.99, 2.07

Abbreviations: CI, confidence interval; RR, rate ratio.

a During refers to year − 1 and 0 in relation to the timing of the life-event exposure.

b After refers to year + 1 to + 3 in relation to the timing of the life-event exposure.

c Before refers to year − 3 and − 2 in relation to the timing of the life-event exposure.

d Rate ratios (RR) and their 95% confidence limits (CI) derived from Poisson regression generalized estimating equation analysis for time periods.

e Adjusted for sex, age, education, marital status, smoking, sedentary lifestyle, obesity, high alcohol intake, depression and sensitivity to stress.

f Additionally adjusted for inhaled corticosteroids and long-acting β_2_-agonists in combination or separate as time dependent variable.

Results from the subgroup analyses are shown in Table 3. Compared with the pre-exposure level, the rate of purchases of inhaled short-acting β_2_-agonists increased in all subgroups of participants during and after high life-event exposure. One exception was related to age; after exposure, the youngest age group showed no increase in the rate of purchases of inhaled short-acting β_2_-agonists. Interestingly, among depressive participants (*n* = 121), the post-exposure increase was exceptionally high, 4.4-fold compared to the pre-exposure level.

**Table 3:** Rate Ratios for Purchases of Inhaled Short-Acting β_2_-Agonists Comparing Different Time Periods by Recent Stressful Life-Event Exposure Levels According to Baseline Characteristics, The Health and Social Support Study in Finland, 1998–2006.

	No stressful life-event exposure				Low stressful life-event exposure				High stressful life-event exposure			
	Time in relation to life-event exposure				Time in relation to life-event exposure				Time in relation to life-event exposure			
	During^a^ vs before^b^		After^c^ vs before^b^		During^a^ vs before^b^		After^c^ vs before^b^		During^a^ vs before^b^		After^c^ vs before^b^	
Characteristic	RR^d^	95% CI	RR^d^	95% CI	RR^d^	95% CI	RR^d^	95% CI	RR^d^	95% CI	RR^d^	95% CI
Sex												
Men	0.89	0.75, 1.06	0.93	0.77, 1.13	0.94	0.70. 1.26	1.02	0.67, 1.57	1.22	0.70, 2.10	1.38	0.59, 3.23
Women	1.00	0.88, 1.15	0.94	0.79, 1.11	0.82	0.72, 1.92	0.85	0.67, 1.08	1.59	1.21, 2.09	1.59	1.17, 2.15
Age group												
20–24	0.84	0.62, 1.13	0.85	0.64, 1.14	0.90	0.63, 1.29	1.06	0.67, 1.67	1.20	0.84, 1.71	0.64	0.35, 1.15
30–34	1.05	0.83, 1.34	1.20	0.87, 1.66	0.91	0.72, 1.16	0.92	0.59, 1.44	1.38	0.75, 2.55	2.29	1.13, 4.64
40–44	0.98	0.76, 1.27	0.94	0.69, 1.28	0.77	0.63, 0.95	0.89	0.68, 1.15	1.66	0.96, 2.86	1.96	1.22, 3.14
50–54	0.99	0.86, 1.15	0.87	0.73, 1.03	0.73	0.59, 0.89	0.70	0.44, 1.11	1.76	1.14, 2.71	2.31	1.45, 3.70
Education												
University	0.97	0.69, 1.37	1.01	0.70, 1.47	0.80	0.64, 1.00	0.79	0.48, 1.32	1.21	0.55, 2.66	1.41	0.60, 3.34
College	0.91	0.74, 1.11	0.84	0.64, 1.11	0.90	0.68, 1.18	1.04	0.71, 1.52	1.57	0.96, 2.56	1.81	1.04, 3.14
Vocational school	0.98	0.79, 1.21	0.94	0.73, 1.21	0.83	0.65, 1.07	0.49	0.34, 0.72	1.13	0.75, 1.70	1.03	0.51, 2.06
Basic	1.04	0.87, 1.25	0.99	0.80, 1.22	0.85	0.71, 1.02	1.04	0.73, 1.46	1.89	1.28, 2.78	2.03	1.25, 3.30
Marital status												
Single/divorced/widowed	0.96	0.79, 1.16	0.96	0.77, 1.21	0.91	0.75, 1.10	0.89	0.64, 1.26	1.31	0.95, 1.83	1.15	0.76, 1.74
Married/cohabiting	0.98	0.86, 1.12	0.92	0.78, 1.09	0.81	0.69, 0.94	0.87	0.67, 1.14	1.57	1.06, 2.31	1.80	1.09, 2.98
Smoking												
Never-smoker	0.92	0.78, 1.07	0.88	0.74, 1.06	0.80	0.61, 1.05	0.89	0.60, 1.30	1.39	0.95, 2.02	1.40	0.97, 2.01
Ex-smoker	1.20	0.95, 1.51	1.16	0.91, 1.49	0.89	0.74, 1.08	0.77	0.55, 1.08	1.23	0.71, 2.16	1.34	0.60, 2.95
Current smoker	0.96	0.79, 1.16	0.96	0.75, 1.23	0.83	0.69, 1.01	0.80	0.57, 1.12	1.65	1.04, 2.62	1.62	0.84, 3.12
Physical inactivity												
No	1.00	0.88, 1.15	0.95	0.81, 1.11	0.81	0.71, 0.93	0.92	0.73, 1.17	1.35	1.04, 1.75	1.28	0.86, 1.90
Yes	0.90	0.77, 1.07	0.90	0.72, 1.13	0.99	0.73, 1.34	0.77	0.51, 1.18	1.97	0.84, 4.61	2.64	1.17, 5.95
Obesity (BMI ≥ 30)^e^												
No	0.98	0.86, 1.12	0.94	0.80, 1.09	0.85	0.74, 0.98	0.87	0.69, 1.10	1.39	1.02, 1.89	1.39	0.93, 2.09
Yes	0.96	0.81, 1.14	0.93	0.74, 1.18	0.76	0.62, 0.92	0.94	0.62, 1.41	1.82	1.28, 2.57	2.19	1.39, 3.47
High alcohol intake^f^												
No	0.97	0.87, 1.08	0.92	0.80, 1.06	0.82	0.72, 0.93	0.87	0.69, 1.10	1.44	1.10, 1.89	1.47	1.02, 2.12
Yes	1.18	0.74, 1.90	1.46	0.83, 2.57	1.07	0.89, 1.29	1.06	0.73, 1.53	2.23	0.66, 7.53	3.76	0.76, 18.50
Depression												
No	0.99	0.88, 1.12	0.96	0.83, 1.11	0.84	0.73, 0.96	0.93	0.73, 1.18	1.34	1.02, 1.75	1.30	0.90, 1.88
Yes	0.90	0.73, 1.12	0.84	0.64, 1.11	0.86	0.66, 1.13	0.70	0.46, 1.07	2.93	1.47, 5.85	4.36	1.75, 10.84
Sensitivity to stress												
No	0.97	0.84, 1.11	0.93	0.80, 1.09	0.77	0.67, 0.90	0.83	0.64, 1.07	1.36	0.97, 1.92	1.29	0.84, 1.97
Yes	0.99	0.83, 1.18	0.92	0.73, 1.15	0.97	0.78, 1.19	0.98	0.67, 1.42	1.56	1,00, 2.41	1.77	0.97, 3.25

Abbreviations: BMI, body mass index; CI, confidence interval; RR, rate ratio.

a During refers to year − 1 and 0 in relation to the timing of the life-event exposure.

b After refers to year + 1 to + 3 in relation to the timing of the life-event exposure.

c Before refers to year − 3 and − 2 in relation to the timing of the life-event exposure.

d Rate ratios (RR) and their 95% confidence limits (CI) derived from Poisson regression generalized estimating equation analysis for time periods.

e BMI was calculated as weight (kg)/height (m^2^).

f High alcohol intake refers to consumption of > 175 g/week for women and > 263 g/week for men.

Appendix 1 presents how many of the 367 participants reporting a recent event at the follow-up survey in 2003 reported the corresponding event also in the 1998 survey. As can be seen, for the most severe events recurrence was non-existing or rare (e.g. only 14% of those who were victims of violence in 2003 reported to have been victims in preceding six months in 1998 also). Highest recurrence was found for ‘breakup of long-term friendship’ (43%), ‘severe financial difficulties’ (40%) and ‘death of a close relative’ (35%), events rated much less severe.

## 4 Discussion

In this population-based 7-year follow-up study on adults with asthma, records of filled prescriptions of inhaled short-acting β_2_-agonists before, during and after exposure to stressful life-events were used as an indicator of asthma symptoms. Among those who had encountered recent highly stressful life events, the rate for inhaled short-acting β_2_-agonists purchases was 1.5 times higher after the life event compared to the pre-event levels. No such increase was observed among those with no or low exposure to stressful life events. These findings support the hypothesis that psychosocial stress may exacerbate asthma symptoms in working-aged adults.

Our results are consistent with the biopsychosocial model of stress, which suggests that stressful life events may alter the psychological, immunological and endocrine systems in ways that lead to the exacerbation of asthma [9], [34]. Moreover, the results of this study are in line with the few studies which investigated the association between stressful life events and the exacerbation of asthma symptoms in adults. A case-control study by Kolbe et al. [19] found that life events were reported more often among patients admitted to hospital with acute asthma compared to a control group of non-hospitalized asthmatics. In a prospective population based study by Wainwright et al. [18] life events experienced in adulthood were associated with increased rates of asthma-related hospital admissions. Although earlier studies provide important insight on asthma exacerbation, hospital admissions for asthma are considered very rare [21], representing only the tip of an iceberg of asthma morbidity. In this study, the finding that the utilization of inhaled short-acting β_2_-agonists varies depending on the extent to which asthmatic adults are exposed to life stress has not been previously reported.

Stress may have an impact on individuals' life management and thus affects asthma self-care and adherence to treatment [34], and increases the risk of inappropriate use of asthma medications [35]. A recent study found that many patients perceived stress was an important determinant of uncontrolled asthma [36]. One indicator of such a development is high use of short-acting bronchodilators with low use of inhaled steroids [35]. As we were able to control in the analysis simultaneous usage of anti-inflammatory medication, poor self-care of asthma is an unlikely explanation for our time-dependent findings.

The strengths of this study are its large sample size and a study design that allowed the determination of temporary order between exposure to recent stressful life events and purchases of asthma medication. The number of filled prescriptions of inhaled short-acting β_2_-agonists and the measurement of asthma were based on national health registers. In Finland, the validity of the national registers has been found to be high [37], reasonably accurate, and highly reliable for epidemiological study purposes [38]. We were also able to control a number of socio-demographic elements and etiological factors of asthma.

A limitation of the study is that filled prescriptions do not equate to actual medication utilization. Because we did not have information on total amounts of drugs per prescription or recommended doses of the asthma treatment, we were not able to use more fine-grained measures such as increased daily doses of inhaled short-acting bronchodilators use. However, short-acting beta-agonist prescription fills can be used as a marker for asthma morbidity [39]. At baseline, the response rate was relatively low (40%), and there may have been differences between respondents and non-respondents regarding the frequency of asthma and recent stressful life events, although no major health-related selection has been detected in a non-response analysis [24]. However, > 80% of the baseline respondents participated in the follow-up survey, and practically all of them (96%) consented to the linking of data from national health registers. Thus, it is unlikely that the longitudinal association between recent stressful life events and asthma exacerbation would be biased due to low participation at baseline. Additionally, due to the applied survey methodology, we did not have any additional information of the reported life events and their stressfulness. However, by using average severity rating instead of individual perception of the severity of the event our measure was not confounded by the consequences of the event for the individual.

## 5 Conclusions

To the best of our knowledge, this is the first study to investigate the longitudinal associations between recent stressful life events and purchase levels in a population sample of adults with asthma at middle age. Our finding that exposure to highly-stressful life events is associated with an increase in inhaled short-acting β_2_-agonists purchases suggest a worsening of asthma symptoms in the aftermath of stressful experiences. These results highlight the potential importance of taking into account psychosocial stress in guiding asthma self-care.

The following is the supplementary data related to this article.

Appendix 1 Specific life events* within the previous 6 months reported in the 2003 survey by the 367 participants and the number of participants reporting the same event within the previous six months in the 1998 survey. Appendix 1

## Competing Interest Statement

All authors have completed the Unified Competing Interest form at http://www.icmje.org/coi_disclosure.pdf and declare that (1) Dr. Kivimäki received support from NordForsk, the Medical Research Council, and the Economic and Social Research Council, during the conduct of the study; (2) authors have no relationships with companies or other competing interests in the past three years that could be perceived to constitute a conflict of interest; (3) spouses, partners, or children of authors have no financial relationships that may be relevant to the submitted work; and (4) authors have no non-financial interests that may be relevant to the submitted work.
