# Supplementary material for: Change in β2-agonist use after severe life events in adults with asthma: A population-based cohort study: Life events and bronchodilator usage among adults with asthma
Source: J Psychosom Res. 2017 Sep;100:46–52. doi: 10.1016/j.jpsychores.2017.07.003 (PMC5556252; doi:10.1016/j.jpsychores.2017.07.003)
Supplement: Appendix 1 — Specific life events* within the previous 6 months reported in the 2003 survey by the 367 participants and the number of participants reporting the same event within the previous six months in the 1998 survey. [file mmc1.docx]

**SUPPLEMENTARY MATERIAL**

Appendix 1— Specific life events* within the previous 6 months reported in the 2003 survey by the 367 participants and the number of participants reporting the same event within the previous six months in the 1998 survey.

| Type of event | Life event in 2003 survey  n | Same event in 1998 survey  n (%) |
| --- | --- | --- |
| Death of own child | 1 | 0 (0) |
| Death of spouse | 2 | 0 (0) |
| Emotional, physical or sexual violence | 14 | 2 (14) |
| Severe illness in a family member | 45 | 12 (27) |
| Death of mother | 11 | - |
| Major increase in marital problems | 83 | 12 (14) |
| Divorce or separation | 19 | 1 (5) |
| Severe conflicts with supervisor | 36 | 9 (25) |
| Severe financial difficulties | 77 | 31 (40) |
| Death of father | 8 | - |
| Severe injury | 15 | 2 (13) |
| Severe conflicts with coworkers | 37 | 6 (16) |
| Miscarriage (own or partner) | 1 | 0 (0) |
| Death of close friend | 35 | 4 (11) |
| Loss of job | 27 | 3 (11) |
| Abortion (own or partner) | 1 | 0 (0) |
| Breakup of long-term friendship | 37 | 16 (43) |
| Death of another close relative | 60 | 21 (35) |
| Unemployment of spouse | 32 | 5 (16) |

*Events listed by decreasing severity rating (for weights, see Vahtera et al. [28])
